# Role of serum C-reactive protein (CRP)/Albumin ratio in predicting the severity of acute pancreatitis: A retrospective cohort

**DOI:** 10.1016/j.amsu.2022.104715

**Published:** 2022-09-21

**Authors:** Syed Jawad Haider Kazmi, Muhammad Talha Zafar, Beenish Fatima Zia, Saleha Rashid Khalid, Vikesh Kumar, Shaesta Tabassum, Ahmed Ali, Nouman Aziz, Noman Ahmed Khan, Kanchan Kumari, Kanza Saleem, Muhammad Sohaib Asghar

**Affiliations:** a Liaquat National Hospital and Medical College, Karachi, Pakistan; b DHQ Hospital Jauharabad, Punjab, Pakistan; c FMH College of Medicine and Dentistry, Lahore, Pakistan; d Civil Hospital, Sukkur, Pakistan; e Fauji Foundation Hospital, Rawalpindi, Pakistan; f Mayo Hospital, Lahore, Pakistan; g Jinnah Sindh Medical University, Karachi, Pakistan; h Dow University of Health Sciences, Karachi, Pakistan

**Keywords:** Pancreas, Severity, ICU, Acute pancreatitis, CRP, Albumin

## Abstract

Acute pancreatitis is a disease with a wide spectrum of severity, complications, and outcome with severe life-threatening complications develop in patients leading to high mortality in severe acute pancreatitis. The rationale of this study is to diagnose the severity of acute pancreatitis using a single test ratio, i.e., CRP/albumin ratio which is a combination of markers for systemic inflammation and nutritional status. All those patients with age group 16–80 years who were diagnosed with acute pancreatitis and admitted subsequently to ICU were included. Severe pancreatitis was determined as CT severity score above 7. About 41% patients out of total 225 had severe pancreatitis. CRP/albumin ratio >4.35 had a sensitivity of 87% and accuracy of 76% to predict acute severe pancreatitis. Elevated CRP/albumin ratio was also associated with complications like multi-organ failure OR: 2.31 [1.3–4.2], duodenal thickening OR: 2.25 [1.2–4.2], and ascites OR: 2.90 [1.5–5.6]. Although, the severity of this elevation varied with different age groups, such non-invasive and readily available parameters should be relied upon admission to risk stratify the patients suffering from pancreatitis. CRP/albumin ratio has higher sensitivity and negative predictive value to predict severe pancreatitis than CRP alone and hence give additional advantage as a prognostic marker, although Delong's test to compare AUROC was indifferent (P-value: 0.22).

## Highlights

• There is a need to diagnose the severity of acute pancreatitis using a single test ratio i.e., CRP/albumin ratio.
• CRP/albumin ratio >4.35 had a sensitivity of 87% and accuracy of 76% to predict acute severe pancreatitis.
• Readily available parameters should be relied upon admission to risk stratify the patients suffering from pa**ncreatitis.**

## 1 Introduction

Acute pancreatitis is one of the severe disease due to acute inflammation of pancreas. Acute pancreatitis is a disease with a wide spectrum of severity, complications, and outcome. Unfortunately, we don't have much data regarding the epidemiology of this disease but the international stats tell us that the incidence of acute pancreatitis in the UK is 56 cases per 100,000 persons per year [1], while in the US over 220,000 hospital admissions annually are attributed to acute pancreatitis [2]. An epidemiologic study that utilized UK and European data demonstrated an increasing incidence in all-cause acute pancreatitis [3]. The incidence of acute pancreatitis was also noted to increase with age [3]. The male population had an incidence that was 10%–30% higher than females [4]. Of all hospital admissions with acute pancreatitis, 20%–30% of patients have a severe course [1], while severe life-threatening complications will develop in 25% of these patients [4]. The mortality in severe acute pancreatitis can be as high as 30% [2], but the overall mortality in acute pancreatitis is estimated to be 5% [1]. Gallstones remain the most common cause for acute pancreatitis. Gallstone-related acute pancreatitis accounts for approximately half of all UK cases, while up to 25% of acute pancreatitis cases can be attributed to alcohol [1]. To date, there is no drug available to treat acute pancreatitis, so most care is supportive. With this limitation, most clinical management guidelines [5,6], emphasize an approach that includes predicting and establishing the severity of acute pancreatitis to triage patients to appropriate levels of care; administering supportive care, including intravenous hydration and enteral nutrition; and treating the underlying cause and complications by appropriate use of urgent endoscopic retrograde cholangiopancreatography (ERCP), early cholecystectomy, targeted use of antibiotics, and interventions for pancreatic fluid collections in the later stages, usually after 4 weeks.

There are various diagnostic tests and criteria scoring systems are available to diagnose acute pancreatitis but none of them has been labeled as gold standard. A list of various inflammatory markers has also been tried for example C-reactive protein (CRP), Procalcitonin, Lactate dehydrogenase, Albumin, etc, have been tried. CRP, now a days being commonly use as a marker of inflammation as its radially available and comparatively economical than others inflammatory markers. C-reactive protein exhibits elevated expression during inflammatory conditions such as rheumatoid arthritis, some cardio-vascular diseases, and infection [7]. As an acute-phase protein, the plasma concentration of CRP deviates by at least 25% during inflammatory disorders [8]. The highest concentrations of CRP are found in serum, with some bacterial infections increasing levels up to 1000-fold [9]. However, when the stimuli ends, CRP values decrease exponentially over 18–20 h, close to the half-life of CRP [10]. CRP plasma levels increase from around 1 μg/mL to over 500 μg/mL within 24–72 h of severe tissue damage such as trauma and progressive cancer [11]. IL-6 is reported to be the main inducer of CRP gene expression, with IL-1 enhancing the effect [12]. However, although IL-6 is necessary for CRP gene induction, it is not sufficient to achieve this alone [13].

The rationale of this study is to diagnose the severity of acute pancreatitis using a single test ratio rather than evaluating through a criteria system like Ranson's Criteria which includes eleven different parameters and more over half of them are sent after 48 h of admission. It will be in great benefit for the patient if we could establish a single test ratio system to predict the severity of acute pancreatitis.

### 1.1 Materials and methods

This retrospective, observational study was conducted at general surgery department of a tertiary care hospital, Karachi. Non-probability consecutive sampling method was used to recruit all the study participants diagnosed with acute pancreatitis on clinical and radiological grounds and admitted to intensive care unit (ICU). CRP/albumin ratio is a combination of markers for systemic inflammation and nutritional status which is calculated by dividing the CRP level (mg/L) with the serum albumin level (g/L). Taking population mean of 2.90 and standard deviation of 3.02 reported by Zhao et al. [14], a sample size of 205 is calculated by using a W.H.O sample size calculator (7.1: Estimating a population mean) in which we used 5% as margin of error, and 95% as confidence interval (CI). All those patients with age group 16–80 years who were diagnosed with acute pancreatitis and admitted subsequently to ICU were included in our study. Those who were having autoimmune diseases, multiple co-morbidities, or any chronic inflammatory conditions were excluded from the study. The study was performed after approval from the IRB board of Liaquat National Hospital and written informed consent from the relevant surgical department of the hospital (UIN: Ref:App.#R.C-LNH-ER-07/2021/73). STROCSS guidelines were adhered while reporting the findings of the study [15].

For data collection, A proforma was designed to have two sections; first section covered demographic details including name (as optional), age (in years), gender, and other clinical (abdominal pain, physical examination findings) and biochemical characteristics (serum amylase/lipase) available from the medical records of the patients. Second section determines the serum albumin and CRP levels correlating with disease severity at admission and comparison with CT severity score. An expert radiology team reported the findings of Computerized Tomography (CT) scan, polygonal descriptive of which is represented in Supplementary Fig. 1; where CT severity score was reported on a scale of 1–10 with 10 being most severe. Score above 7 was determined as severe pancreatitis, while up to 2 was labeled as mild form; and moderate disease is between 3 and 6 severity score. Data was collected after laboratory evaluation is done through serum samples for CRP and albumin sent for the enrolled patients at admission. CRP was analyzed via Cobas (C-311) using immunoturbidimetric assay (Roche/Hitachi Cobas C systems).

After collection of data the analyses was conducted by using Statistical Package for Social Science (IBM SPSS) software, version 25.0 (Armonk, NY, USA). The data was checked for normality via the Shapiro-Wilk test and plotting a histogram. The Chi-square test was used to compare categorical variables, and if found limited, Fisher's exact test will be applied. Receiver operating characteristic (ROC) curves was used to evaluate the diagnostic performance of CRP/albumin ratio in determining the severity of pancreatitis. An optimum cut-off value was obtained for significance against the area under the curve (AUC). ROC analysis was used to calculate the appropriate sensitivity, specificity, positive predictive value, negative predictive value, positive likelihood ratio, negative likelihood ratio and diagnostic accuracy of CRP/Albumin ratio in determining severe pancreatitis against a CT severity score. Sensitivity refers to the probability of a positive test, conditioned on truly being positive (TP), calculated as TP/TP + FN; Specificity refers to the probability of a negative test, conditioned on truly being negative (TN), calculated as TN/TN + FP; and diagnostic accuracy: the proportion of correct diagnosis found by a diagnostic test, calculated as TP + TN/TN + FP + FN + TP was given priority. Lastly, Delong's was applied for statistical comparisons between CRP/albumin ratio and CRP along as predictive marker of severity of pancreatitis. MedCalc Statistical Software Ltd (free trial version) was used for applying Delong's test [16].

## 2 Results

The study included 225 patients diagnosed with acute pancreatitis with 60% males, and 40–50 years of age group more frequently represented (27%). A total of 93 individuals had severe pancreatitis (CT severity score≥7). Other complications included peri-pancreatic fluid (94%), ascites (78%), pleural effusion (73%), multi-organ failure (72.4%), and duodenal swelling/thickening (25%) as shown in Table 1. All of these complications were expectedly higher in severe pancreatitis as shown in Table 2.

**Table 1:** Baseline clinical characteristics and radiological findings of the study inclusions (n = 225).

Variables	Characteristics	Frequency	Percentage
Age (in years)	16–20	N = 14	6.2%
	21–30	N = 39	17.3%
	31–40	N = 46	20.4%
	41–50	N = 61	27.1%
	51–60	N = 39	17.3%
	61–80	N = 26	11.6%
Gender	Male	N = 136	60.4%
	Female	N = 89	39.6%
CT severity score	mild 0-2	N = 13	5.8%
	moderate 3-6	N = 119	52.9%
	severe 7-10	N = 93	41.3%
Multiorgan failure (MOF)	Yes	N = 163	72.4%
	No	N = 62	27.6%
Duodenal thickening	Swollen	N = 57	25.3%
	Not swollen	N = 168	74.7%
Peri-Pancreatic fluid	Present	N = 211	93.8%
	Absent	N = 14	6.2%
Ascites	Present	N = 175	77.8%
	Absent	N = 50	22.2%
Pleural effusion	Present	N = 165	73.3%
	Absent	N = 60	26.7%

CT: computerized tomography.

**Table 2:** Categorical comparisons of pancreatitis severity and study variables (n = 225).

CT severity score		Mild (0–2) N = 13	Moderate (3–6) N = 119	Severe (7-10) N = 93	p-value
Multiorgan failure (MOF)	No	13 (100.0%)	42 (35.3%)	7 (7.5%)	<0.001^b^
	Yes	0 (0.0%)	77 (64.7%)	86 (92.5%)	
Duodenal thickening	No	13 (100.0%)	97 (81.5%)	58 (62.4%)	0.001^b^
	Yes	0 (0.0%)	22 (18.5%)	35 (37.6%)	
Peri-Pancreatic fluid	No	4 (30.8%)	7 (5.9%)	3 (3.2%)	0.005^b^
	Yes	9 (69.2%)	112 (94.1%)	90 (96.8%)	
Ascites	No	12 (92.3%)	32 (26.9%)	6 (6.5%)	<0.001^a^
	Yes	1 (7.7%)	87 (73.1%)	87 (93.5%)	
Pleural effusion	No	12 (92.3%)	37 (31.1%)	11 (11.8%)	<0.001^b^
	Yes	1 (7.7%)	82 (68.9%)	82 (88.2%)	
CRP/Alb ratio	<4.348	12 (92.3%)	79 (66.4%)	17 (18.3%)	<0.001^a^
	>4.348	1 (7.7%)	40 (33.6%)	76 (81.7%)	

CRP: c-reaction protein; Alb: albumin.

a Chi-square test.

b Fisher's Exact test.

**Fig. 1:**
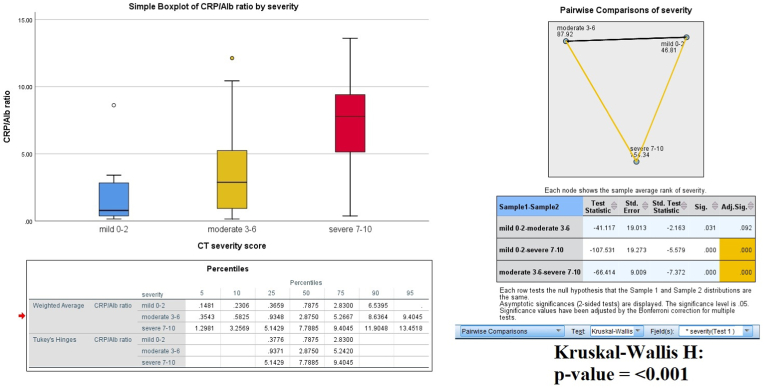
Box-plots representing CRP/albumin ratios among severity of pancreatitis along with pair-wise comparisons with Post-hoc Bonferroni method applied.

**Fig. 2:**
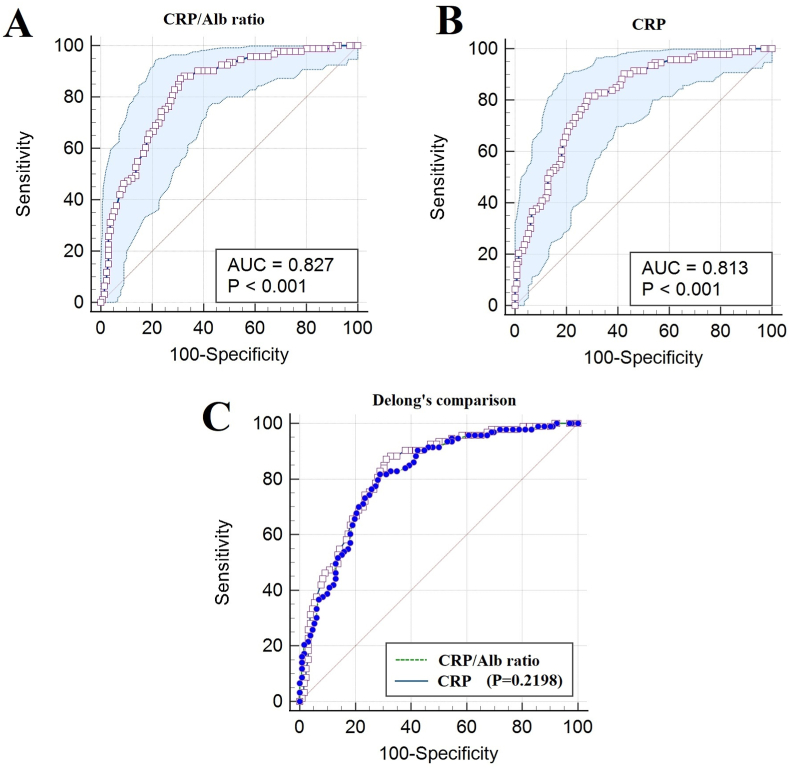
Area under the receiver operating characteristics (AUROC) curves for CRP/albumin ratio (A), CRP alone (B), and both A & B combined (C).

Median CRP/albumin ratio was 4.38 [1.41–8.49] for whole cohort and 7.79 [5.14–9.40] for severe pancreatitis group as shown in Fig. 1. Pair-wise comparisons revealed greater difference between mild to severe, moderate to severe pancreatitis (Kruskal Wallis P-value: <0.001). ROC analysis for CRP/albumin ratio is shown in Fig. 2 and Table 3. Optimum cut-off value of 4.35 was determined for CRP/albumin ratio via highest Youden index at which the sensitivity to predict severe pancreatitis was 87% as compared to 82% for CRP alone. Although this comparison is not statistically indifferent at Delong's test (P-value: 0.22) with similar diagnostic accuracy of 76% vs 74%, however the ratio had higher AUC (0.827) and higher negative predictive value (88%). Interestingly, patients with elevated CRP/albumin ratio had higher odds with complications like multi-organ failure 2.31 [1.3–4.2], duodenal thickening 2.25 [1.2–4.2], and ascites 2.90 [1.5–5.6] as shown in Table 4.

**Table 3:** Receiver Operating Characteristics (ROC) analysis CRP/Alb ratio for severity of pancreatitis.

Variable	CRP/Alb ratio	CRP alone	Delong's comparison
AUC	0.827	0.813	–
95% CI	0.771–0.874	0.756–0.862	–
Cut-off value	4.348	10.99	–
Standard error	0.0275	0.0285	0.0111
Youden's index	0.5604	0.5293	–
Sensitivity	87.10%	81.72%	–
95% CI	78.5–93.2%	72.4–89.0%	–
Specificity	68.94%	71.21%	–
95% CI	60.3–76.7%	62.7–78.8%	–
+ likelihood ratio	2.80	2.84	–
95% CI	2.15–3.66	2.13–3.77	–
– likelihood ratio	0.19	0.26	–
95% CI	0.11–0.32	0.16–0.40	–
PPV	66.4%	65.0%	–
NPV	88.3%	84.3%	–
Accuracy	76.4%	74.2%	–
p-value	<0.0001	<0.0001	0.2198
z-statistic	–	–	1.227
Difference between area	–	–	0.0136
95% CI	–	–	−0.00810–0.0352

ROC: receiver operating characteristics; AUC: area under the curve; CI: confidence interval; PPV: positive predictive value; NPV: negative predictive value; CRP: c-reactive protein; Alb: Albumin.

**Table 4:** Multivariable regression for pancreatitis complications with elevated CRP/Alb ratio^a^.

Variables	OR	95% CI	p-value	aOR	95% CI	p-value
Multiorgan failure (MOF)	2.310	1.266–4.216	**0.006**	3.711	1.087–12.674	**0.036**
Duodenal thickening	2.253	1.202–4.224	**0.011**	2.062	1.078–3.945	**0.029**
Peri-Pancreatic fluid	2.036	0.660–6.280	0.216	1.446	0.367–5.700	0.598
Ascites	2.900	1.491–5.643	**0.002**	2.393	1.005–5.698	**0.049**
Pleural effusion	1.467	0.810–2.656	0.206	0.303	0.085–1.073	0.064

CRP: c-reaction protein; Alb: albumin; OR: odds ratio; aOR: adjusted odds ratio; CI: confidence interval.

a Dependent variable = CRP/Alb ratio >4.348.

An attempt was also made to decipher age-based thresholds for deranged CRP/albumin ratio in acute pancreatitis as shown in Supplementary Table 1. Higher measures of distribution were noticed among bimodal age groups (16–30 years and then 41–60 years), while 31–40 years and extreme age group (61–80 years) had the lowest 95th percentiles for CRP/albumin ratio.

## 3 Discussion

Previous studies have shown CRP/albumin ratio as an important prognostic marker in acute pancreatitis. Mustafa K. et al., in 2017, concluded in his study that the CRP/albumin ratio is a novel but promising, easy-to-measure, repeatable, non-invasive inflammation-based prognostic score in acute pancreatitis [17]. Previously, CRP/albumin ratio has been utilized to predict the prognosis in emergency surgical patients undergoing intensive care [18]. While, one study conducted on animals determined role of CRP/albumin ratio in survival of acute pancreatitis [19]. Kalafat et al. determined a cut-off value for CRP/albumin ratio of 1.08 as highly specific (97%) for diagnosis of acute pancreatitis [20]. Another study determined a direct correlation of Ranson's score with CRP/albumin ratio. Zhao et al. found CRP/Albumin ratio independently associated with mortality in acute pancreatitis patients [14].

Some studies also shown weak association of CRP levels alone at admission and 48 h after hospital stay with complicated acute pancreatitis [21]. While modified CT severity index was considered most predictive for severity of the disease. But Li Y et al. found significant association of CRP levels with mortality [22]. While Han S et al. studied CRP and albumin levels separately and found not only significant correlations with Ranson's score but also the accuracy of Ranson's scale was improved with elevated CRP and decreased albumin levels [23]. This justified our analysis of predicting severity with CRP/Albumin ratio rather than CRP levels alone. Lastly, CRP/albumin ratio was considered a good prognostic marker in predicting severe acute pancreatitis with significantly moderate correlations with all severity scores and hospitalization period [24]. Similar findings were reported Yilmaz and his colleagues [25]. Even accounting for other inflammatory markers like Ferritin, CRP/albumin ratio stands out in predicting severity of the disease and mortality in acute pancreatitis [26].

There are several important factors that could have contributed to limitations of this study. This is a single-center evaluation with a small sample size hence limiting the generalizability of the results. The external validity is also compromised with lack of patient's data with respect to their prognosis, ICU data with parameters (like APACHE-II), further management and disease outcomes. While, internal validity is compromised with the introduction of collider bias when two variables have the same denominator i.e., CRP levels; as part of the ratio also increases the likelihood of comparative ROC analysis been insignificant.

## 4 Conclusion

Although, the statistical difference between CRP/albumin ratio and CRP levels alone isn't much, but overall, the sensitivity and accuracy are increased to predict severe pancreatitis by bringing albumin into the picture. Such non-invasive and readily available parameters should be relied upon admission to risk stratify the patients suffering from pancreatitis.

## Ethical approval statement

Ethical approval was taken in this study from institutional review board of Liaquat National Hospital and Medical College (Ref:App.#R.C-LNH-ER-07/2021/73).

## Sources of funding

None.

## Author contributions

S.J.H.K, and M.S.A, conceived the idea; K.K, K.S, M.T.Z, B.F.Z, and S.R.K, collected the data; V.K, and Z.U.J, analyzed and interpreted the data; A.A, M.S.A, and S.J.H.K, did write up of the manuscript; and finally, M.S.A reviewed and revised the manuscript for intellectual content critically. All authors approved the final version of the manuscript.

## Registration of research studies

Name of the registry: Liaquat National Hospital and Medical College.

Unique Identifying number or registration ID: Ref:App.#R.C-LNH-ER-07/2021/73.

Hyperlink to your specific registration (must be publicly accessible and will be checked).

## Guarantor

Syed Jawad Haider Kazmi.

## Patient consent

Consent to participate from the patients was waived and not required due to retrospective nature of the data collection.

## Provenance and peer review

Not commissioned, externally peer reviewed.

## Data availability statement

Data can be made available on request from corresponding author.

## Declaration of competing interest

The authors have no conflict of interest.

## Appendix A Supplementary data

Multimedia component 2 Multimedia component 2

Supplementary Fig. 1 Frequency polygon for reporting CT severity score among the study population (n = 225). Supplementary Fig. 1
