# Supplementary material for: Role of serum C-reactive protein (CRP)/Albumin ratio in predicting the severity of acute pancreatitis: A retrospective cohort
Source: Ann Med Surg (Lond). 2022 Sep 21;82:104715. doi: 10.1016/j.amsu.2022.104715 (PMC9577824; doi:10.1016/j.amsu.2022.104715)
Supplement: Multimedia component 1 [file mmc1.doc]

| **Supplementary Table 1:** Age-based stratification for distribution of CRP/Alb ratio among the study population. | | | | | | | |
| --- | --- | --- | --- | --- | --- | --- | --- |
| **CRP/Alb ratio** | **For whole cohort (n=225)** | | | | | | |
|  | Total (n=225) | Age 16-20 (n=14) | Age 21-30 (n=39) | Age 31-40 (n=46) | Age 41-50 (n=61) | Age 51-60 (n=39) | Age 61-80 (n=26) |
| Median | 4.38 | 6.96 | 4.64 | 4.36 | 4.38 | 4.71 | 2.85 |
| 25^th^ quartile | 1.41 | 3.76 | 1.25 | 1.18 | 2.10 | 1.40 | 1.04 |
| 75^th^ quartile | 8.49 | 10.61 | 8.34 | 7.43 | 8.64 | 8.74 | 6.18 |
| 95^th^ quartile | 11.77 | 12.45 | 11.93 | 9.40 | 10.47 | 12.25 | 8.99 |
| Mean | 5.07 | 6.74 | 5.23 | 4.72 | 5.15 | 5.43 | 3.81 |
| Standard deviation | 3.66 | 4.38 | 3.76 | 3.25 | 3.66 | 3.96 | 3.10 |
| Minimum | 0.14 | 0.38 | 0.15 | 0.35 | 0.34 | 0.14 | 0.15 |
| Maximum | 13.60 | 13.45 | 13.44 | 12.12 | 13.60 | 13.59 | 10.43 |
| **CRP/Alb ratio** | **For severe pancreatitis group (n=93)** | | | | | | |
|  | Total (n=93) | Age 16-20 (n=8) | Age 21-30 (n=19) | Age 31-40 (n=20) | Age 41-50 (n=30) | Age 51-60 (n=10) | Age 61-80 (n=6) |
| Median | 7.79 | 10.00 | 7.04 | 7.43 | 7.78 | 8.96 | 6.80 |
| 25^th^ quartile | 5.14 | 7.17 | 4.02 | 5.61 | 5.29 | 5.98 | 5.57 |
| 75^th^ quartile | 9.40 | 11.38 | 8.72 | 9.07 | 9.32 | 10.18 | 7.17 |
| 95^th^ quartile | 13.45 | 12.91 | 12.25 | 9.97 | 12.84 | 13.53 | 7.83 |
| Mean | 7.41 | 9.15 | 6.66 | 7.16 | 7.45 | 8.71 | 5.86 |
| Standard deviation | 3.23 | 3.43 | 3.93 | 2.48 | 3.13 | 3.36 | 2.53 |
| Minimum | 0.37 | 3.56 | 0.38 | 2.40 | 1.41 | 3.95 | 1.04 |
| Maximum | 13.60 | 13.45 | 13.44 | 12.12 | 13.60 | 13.59 | 8.04 |
| CRP: c-reactive protein; Alb: albumin. | | | | | | | |
