# Supplementary figures and images for: Role of serum C-reactive protein (CRP)/Albumin ratio in predicting the severity of acute pancreatitis: A retrospective cohort
Source: Ann Med Surg (Lond). 2022 Sep 21;82:104715. doi: 10.1016/j.amsu.2022.104715 (PMC9577824; doi:10.1016/j.amsu.2022.104715)

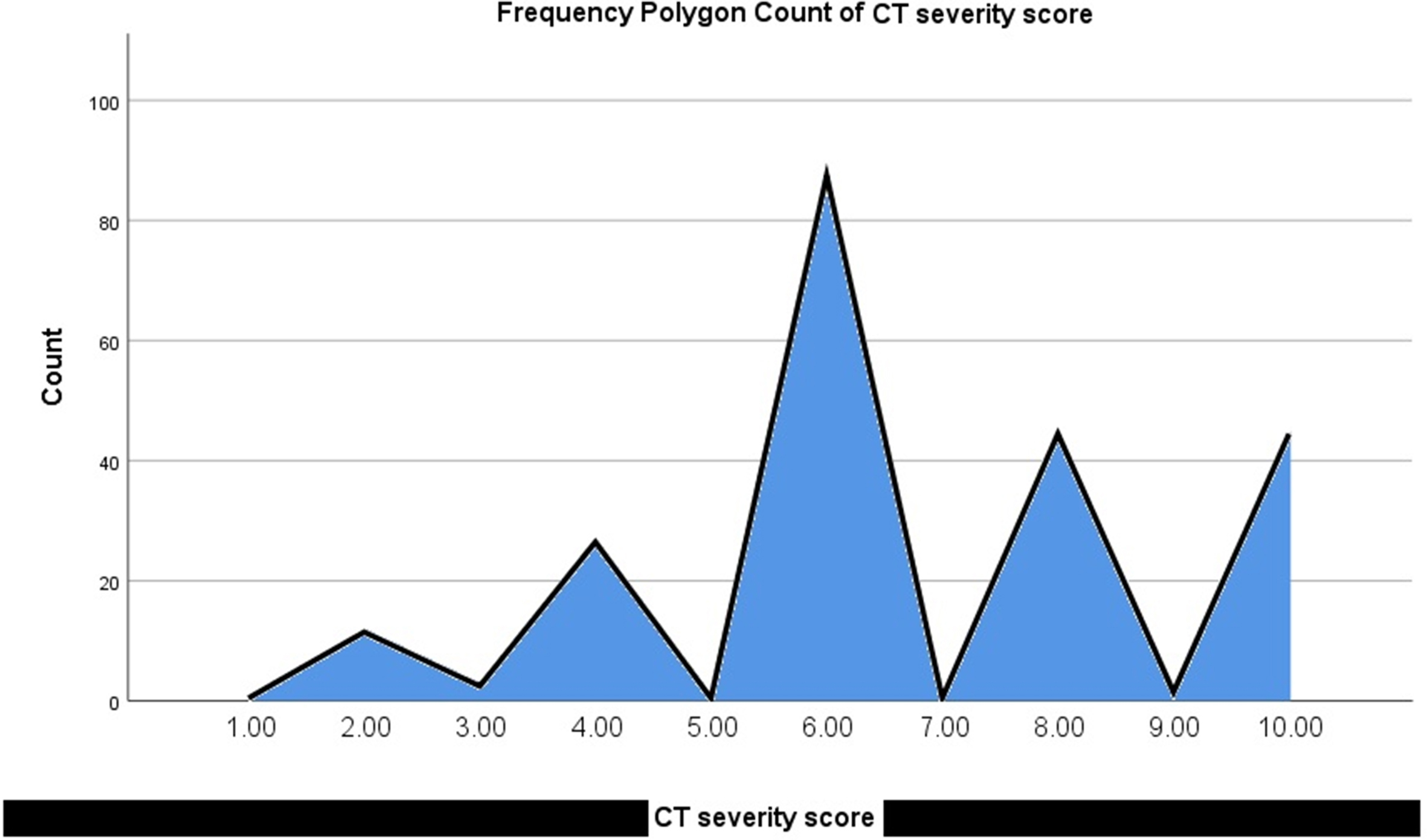

Supplement: Supplementary Fig. 1 — Frequency polygon for reporting CT severity score among the study population (n = 225). [file mmcfigs1.jpg]
